# Measuring the Cognitive Attentional Syndrome in Cardiac Patients With Anxiety and Depression Symptoms: Psychometric Properties of the CAS-1R

**DOI:** 10.3389/fpsyg.2019.02109

**Published:** 2019-09-18

**Authors:** Cintia L. Faija, David Reeves, Calvin Heal, Lora Capobianco, Rebecca Anderson, Adrian Wells

**Affiliations:** ^1^Division of Nursing, Midwifery & Social Work, Faculty of Biology, Medicine and Health, Manchester Academic Health Science Centre, The University of Manchester, Manchester, United Kingdom; ^2^Manchester Academic Health Science Centre, NIHR School for Primary Care Research, The University of Manchester, Manchester, United Kingdom; ^3^Faculty of Biology, Medicine and Health, Centre for Biostatistics, Manchester Academic Health Science Centre, The University of Manchester, Manchester, United Kingdom; ^4^Faculty of Biology, Medicine and Health, School of Psychological Sciences, Manchester Academic Health Science Centre, The University of Manchester, Manchester, United Kingdom; ^5^Greater Manchester Mental Health NHS Foundation Trust, Manchester Royal Infirmary, Manchester, United Kingdom

**Keywords:** cardiac, cognitive attentional syndrome, anxiety, depression, metacognitive therapy

## Abstract

Metacognitive Therapy (MCT) is a recent treatment with established efficacy in mental health settings. MCT is grounded in the Self-Regulatory Executive Function (S-REF) model of emotional disorders and treats a negative perseverative style of thinking called the cognitive attentional syndrome (CAS), thought to maintain psychological disorders, such as anxiety and depression. The evaluation of effective psychological therapies for anxiety and depression in chronic physical illness is a priority and research in this area depends on the suitability and validity of measures assessing key psychological constructs. The present study examined the psychometric performance of a ten-item scale measuring the CAS, the CAS-1R, in a sample of cardiac rehabilitation patients experiencing mild to severe symptoms of anxiety and/or depression (*N* = 440). Participants completed the CAS scale, the Hospital Anxiety and Depression Scale and the Metacognitions Questionnaire 30 (MCQ-30). The latent structure of the CAS-1R was assessed using confirmatory factor analyses (CFA). In addition, the validity of the measure in explaining anxiety and depression was assessed using hierarchical regression. CFA supported a three-factor solution (i.e., coping strategies, negative metacognitive beliefs and positive metacognitive beliefs). CFA demonstrated a good fit, with a CFI = 0.988 and an RMSEA = 0.041 (90% CI = 0.017–0.063). Internal consistency was acceptable for the first two factors but low for the third, though all three demonstrated construct validity and the measure accounted for additional variance in anxiety and depression beyond age and gender. Results support the multi-factorial assessment of the CAS using this instrument, and demonstrate suitability for use in cardiac patients who are psychologically distressed.

## Introduction

Coronary heart disease is the leading cause of death for adult men and women worldwide in developed countries (World Health Organization, 2017). Prevalence of anxiety and depression among patients with cardiovascular disease is up to 3-fold higher than in the general population (Thombs et al., 2008; Tully et al., 2014). Anxiety and depression have been associated with adverse outcomes, such as increased risk of mortality and increased risk of future cardiac problems, poorer quality of life, poorer treatment adherence, and greater health care use (Thombs et al., 2008; Frasure-Smith and Lesperance, 2010; Palacios et al., 2018). Furthermore, anxiety and depression were found to be risk factors for cardiac comorbidity (Halaris, 2009). Following a cardiac event or procedure, patients are offered cardiac rehabilitation (CR) to improve health outcomes and prevent future cardiac problems (Lesperance and Frasure-Smith, 2000). The European Association of Preventive Cardiology has emphasized that symptoms of anxiety and depression in heart disease patients play a key role in the success of CR programmes (Piepoli et al., 2014). CR programmes usually include elements aiming to influence psychological and/or psychosocial outcomes. However, an audit of CR in the United Kingdom (2018) showed that when patients enter the CR programme, 27.5% experienced borderline or clinical levels of anxiety and after the programme 21% remained in those categories. In relation to depression, 18% experienced borderline or clinical levels of depression before starting CR and 12% continued to report depression afterwards (British Heart Foundation, 2018). The variation of improvement across CR programmes ranged from −13 to 43.6% for anxiety and from −12.5 to 36.4% for depression, suggesting that some patients got worse and a substantial number of them did not achieve the national average change in levels of anxiety and/or depression after CR (British Heart Foundation, 2018).

A Cochrane review including 24 randomized controlled trials evaluating effectiveness of psychological interventions vs. usual care, administered by trained staff among coronary heart disease patients, reported small to moderate improvements in depression (*d* = 0.21) and anxiety (*d* = 0.25) (Whalley et al., 2011). Furthermore, other studies highlighted that attempts to treat psychological distress in cardiac patients have shown non-significant improvements in anxiety and depression (Dickens et al., 2013; Reid et al., 2013; Jiang et al., 2017). The National Institute for Health and Care Excellence (NICE) currently recommends cognitive behavioral therapy (CBT) as the first-line treatment for anxiety (NICE, 2014) and depression (NICE, 2016). The CBT model suggests that anxiety and depression are maintained by cognitive distortions and unhelpful behaviors; CBT adopts a range of strategies to challenge the content of negative automatic thoughts to overcome negative emotions (Beck, 1967, 1976). A recent meta-analysis including 12 randomized controlled trials of CBT in cardiac patients showed small to moderate effects in improving anxiety (*d* = 0.34) and depression (*d* = 0.35) compared mainly to usual care (Reavell et al., 2018). Evidence suggests that there is considerable scope for improving outcomes of psychological interventions aimed at reducing anxiety and/or depression in the cardiac population. This has led to a recent National Institute for Health Research (NIHR) funded research programme, called PATHWAY, to examine the effects of a newer form of treatment, metacognitive therapy (MCT: Wells, 2009). A recent meta-analysis evaluating MCT has shown that this therapeutic approach is highly effective in adult mental health settings (Normann and Morina, 2018). The treatment is based on the Self-Regulatory Executive Function (S-REF) model (Wells and Matthews, 1994, 1996). The S-REF model proposes that a particular style of responding to negative thoughts called the cognitive attentional syndrome (CAS) contributes to and maintains emotional disorders and symptoms of distress (e.g., anxiety, depression) (Wells and Matthews, 1994, 1996). The CAS consists of “a perseverative thinking style that takes the form of worry and rumination, attentional focusing on threat, and unhelpful coping behaviors (e.g., thought suppression, avoidance, substance use)” (Wells, 2009, p. 10). It is problematic because it maintains negative processing and a sense of current and future threat. The CAS is thought to be caused, in part, by metacognitive beliefs that individuals hold about their thinking, such as the belief that worrying is useful for coping with threats and the belief that worrying is uncontrollable and dangerous. These positive and negative metacognitions give rise to extended negative thinking patterns that maintain awareness of threat and consequent emotional distress. In sum, the CAS locks the individual into prolonged negative emotional experiences and interferes with adaptive self-regulation, leading to feelings of hopelessness, loss of subjective control over cognition and emotion, and lack of flexibility in implementing alternative thinking styles (Wells, 2009). In contrast to CBT that challenges the content of negative thoughts, MCT aims to interrupt the CAS and challenges metacognitive beliefs using techniques, such as the metacognitive Socratic dialogue, detached mindfulness and attention training techniques (Wells, 2009).

Although trait measures exist to assess metacognitions (Wells and Cartwright-Hatton, 2004) and some dimensions of thinking style (Wells, 1994, 2009; Nolen-Hoeksema, 2000; Ehring et al., 2011), for purposes of assessment of change in treatment it is useful to measure these factors as state variables and to have one instrument that can assess all elements/factors of the CAS simultaneously. Then, changes in these key mechanisms can be monitored over time in an easy and accessible way. With this objective in mind, Wells developed the Cognitive Attentional Syndrome Scale-1 (CAS-1) (Wells, 2009) which includes items to assess maladaptive coping strategies (e.g., dwelling, worrying, focusing attention on threat, avoidance, use of alcohol/drugs) to cope with negative thoughts, and underlying negative and positive metacognitive beliefs.

The CAS-1 (Wells, 2009) has been used by clinicians delivering MCT to measure weekly changes in the CAS, and it has been recently used in research among a non-clinical sample (Fergus et al., 2012; Fergus and Scullin, 2017) and a clinical sample with primary mood or anxiety disorder (Fergus et al., 2013). The CAS-1 has been recently used in medical samples (e.g., cancer, multiple sclerosis, cardiac) (Cook et al., 2015; Heffer-Rahn and Fisher, 2015; Fisher et al., 2017; Wells et al., 2018a,b) but the factorial structure of it has not been explored.

The NIHR UK recently funded a programme of research to examine MCT for anxiety and depression in CR patients (trial protocols: Wells et al., 2018a,b). As MCT aims to target the CAS, it is necessary to assess and monitor changes in this construct. The ethical committee strongly advised reducing respondent burden in the context of the PATHWAY research study. Therefore, the CAS-1 (Wells, 2009) was revised, resulting in a shortened version of 10-items. The revised version of the instrument, Cognitive Attentional Syndrome Scale-1 Revised (CAS-1R) differs from the CAS-1 (Wells, 2009) in using a reduced number of items to assess the CAS and a different rating scale (0–100) rather than (0–8) for all the items. Six items were identified for removal from the original scale by its developer (Adrian Wells), based on clinical and research expertise using the scale. The goal was to produce a revised version incorporating the minimum number of items required to reliably assess all important elements of the CAS (e.g., worry/rumination and other coping strategies, and metacognitive beliefs).

### Aims

**Figure 1:**
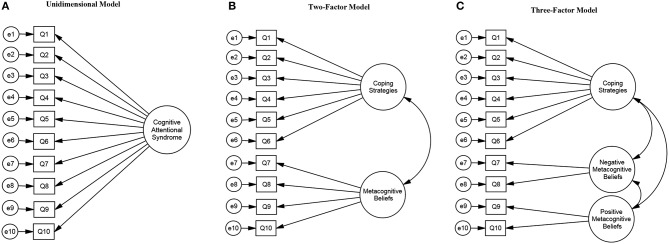
Cognitive Attentional Syndrome Scale-1 Revised (CAS-1R) models.

The aim of the present study was to investigate the psychometric properties of the CAS-1R in cardiac patients with co-morbid anxiety and/or depression. Specifically, we investigated four theoretically based models of the latent structure of the measure. Each of the structural models was derived from the S-REF model (Wells and Matthews, 1994, 1996). As shown in Figure 1 each model introduces incremental refinement to the factor structure. A unidimensional model (Figure 1A) was set as the baseline model in which all items load on a general factor called CAS making no distinctions between sub-components. The two-factor model (Figure 1B) differentiates between proximal and distal causative mechanisms of emotional disorders. Specifically, proximal mechanisms that maintain negative emotional experiences are included in the factor named Coping Strategies, which combines conceptual and attentional processes in the form of worry, rumination, focusing attention on threat and also strategies, such as thought suppression, avoidance (emotional and cognitive) and alcohol use. Distal mechanisms underlying anxiety and depression disorders are the metacognitive beliefs that people hold about their thinking, thus, the second factor in this model was labeled metacognitive beliefs. The three-factor model (Figure 1C) includes a separation between negative and positive metacognitive beliefs. From a theoretical and clinical perspective it is relevant to examine these two content domains of metacognitive beliefs separately. Specifically, negative metacognitive beliefs lead individuals into a sense of threat from thoughts themselves, unhelpful types of mental control or diminished control attempts, whilst positive metacognitive beliefs contribute to worrying and rumination as strategies to cope with distressing negative thoughts (Wells, 2009). Empirical evidence has shown that negative metacognitive beliefs are a strong predictor of anxiety and depression in mental health, physical health, student and community samples (Sun et al., 2017) and positive metacognitive beliefs are associated with rumination in depression (Papageorgiou and Wells, 2003). Thus, maintaining a differentiation between positive and negative metacognitive beliefs might help to identify whether different metacognitive beliefs predict anxiety, and/or depression symptoms. Finally, a bi-factor model consisting of the same three factors depicted in Figure 1C with the addition of a general factor contributing to all the individual items was hypothesized, in order to explore if a general factor would carry additional information beyond that conveyed by the three factors alone.

The primary aim of the study was to identify which factor structure fitted the underlying data best in order to evaluate theoretically derived construct validity of the instrument among a CR sample experiencing symptoms of anxiety and/or depression. The secondary aims of the study were: (i) to assess convergent and discriminant validity of the CAS-1R; (ii) to examine whether the CAS-1R explains variance in anxiety and/or depressive symptoms in cardiac patients after controlling for age and gender. Gender was controlled following evidence highlighting that depression and anxiety disorders are more prevalent in women than in men (Nolen-Hoeksema, 2001; Simonds and Whiffen, 2003; McLean and Anderson, 2009; Jalnapurkar et al., 2018). Age was controlled because anxiety and depression varies across the lifespan (Jorm, 2000; Lenze and Wetherell, 2011). Moreover, research studies examining the S-REF model and effectiveness of MCT using other measures of metacognition, such as the MCQ-30 (Wells and Cartwright-Hatton, 2004) have controlled for age and gender (e.g., Yilmaz et al., 2011; Hjemdal et al., 2013; Ryum et al., 2018). It is therefore important to explore if the results are consistent with previous findings when using a measure that assesses different elements of the CAS and not only metacognitive beliefs.

## Materials and Methods

### Ethics Statement

This study draws on data collected under a five years programme of research funded by the National Institute for Health Research (NIHR) and sponsored by Greater Manchester Mental Health NHS Foundation Trust. The research programme is called PATHWAY and the Chief Investigator is Professor Adrian Wells. The aim of the programme is to improve effectiveness of psychological interventions for anxiety and depression in CR services. The psychological intervention delivered is MCT (Wells, 2009). Ethical approval for the PATHWAY programme has been granted by the NHS Research Ethics Committee, UK. The Group-MCT Trial (Wells et al., 2018a) received ethical approval from Preston Research Ethics Committee (Ref: 15/NW/0163) and the Home-based MCT Feasibility Trial (Wells et al., 2018b) received ethical approval from the North West-Greater Manchester West Research Ethics Committee (Ref: 16/NW/0786).

### Participants and Procedure

Participants were recruited from CR services at seven National Health Services (NHS) Trusts in the North-West of England. Participants were invited to take part in the PATHWAY Programme if they met the eligibility criteria presented on Table 1. In the present study, anxiety and/or depression symptoms were defined by a score of 8 or more on either of the subscales of the Hospital Anxiety and Depression Scale (HADS) which corresponds to at least a mild category (HADS; Zigmond and Snaith, 1983). In a general population, a score of 8 provides 82% sensitivity and 74% specificity for detecting major depressive disorder, and 78% sensitivity and 74% specificity for identifying generalized anxiety disorder (Brennan et al., 2010). Fifty-three percent of the patients invited to take part in the PATHWAY programme agreed to participate.

**Table 1:** Participant's eligibility: inclusion and exclusion criteria.

**INCLUSION CRITERIA**
(i) Patients were referred to the cardiac rehabilitation services
(ii) A score of ≥8 on the depression and/or anxiety subscale of the Hospital Anxiety and Depression Scale (Zigmond and Snaith, 1983)
(iii) Minimum of 18 years old
(iv) Competent level of English language skills
**EXCLUSION CRITERIA**
(i) Cognitive impairment precluding informed consent or ability to participate
(ii) Acute suicidality
(iii) Active psychotic disorder
(iv) Current drug/alcohol abuse
(v) Concurrent psychological intervention for emotional distress that is not part of usual care
(vi) Antidepressant or anxiolytic medications initiated in the previous 8 weeks
(vii) Life expectancy of <12 months

Patients meeting the inclusion criteria were identified by NHS CR staff that also provided an invitation flier and the patient information sheet to interested patients. All eligible and interested patients were asked to provide written informed consent prior to participating in the study and were then asked to complete the study questionnaires at baseline, 4 and 12 months follow up. Data for the present study include baseline measures only (before receiving any treatment).

The sample consisted of 440 participants experiencing mild to severe symptoms of anxiety and/or depression referred to CR services. The sample mean age was 60.24 (*SD* = 10.76, age range from 27 to 87), the majority of the sample were male (65.5%), white (90.8%), with almost half of the participants married (48.9%), and 78% reported having achieved an educational qualification (e.g., GCSE, diploma, degree).

### Measures

#### The Cognitive Attentional Syndrome Scale-1 Revised (CAS-1R) (Wells, 2015)

The original CAS-1 questionnaire is a 16-item self-report questionnaire developed to assess the different elements of the cognitive attentional syndrome (Wells, 2009, p. 268). The CAS-1 has demonstrated adequate internal consistency (Cronbach's Alpha between 0.78 and 0.86) (Fergus et al., 2012, 2013) and has shown good convergent validity with a measure assessing psychological inflexibility (*r* = 0.63) (Fergus et al., 2013). The CAS-1 was revised by shortening it to 10-items and changing the response scale to increase consistency of responses across items. Examples of the CAS-1R items are: “How much time in the last week have you found yourself dwelling on or worrying about problems [e.g., health, family, finances]?,” “How much do you believe that worrying or dwelling on thoughts is uncontrollable?” Items are rated based on the past 7 days on an 11-point response scale ranging from 0 (none of the time/not at all true) to 100 (all of the time/completely certain this is true) in steps of 10. This is the first study assessing the factorial structure of the CAS-1R measure.

#### The Hospital Anxiety and Depression Scale (HADS; Zigmond and Snaith, 1983)

The HADS is a 14-item self-report scale assessing anxiety (7 items) and depression (7 items). Respondents rate the items based on the past 7 days using a four-point scale (from 0 to 3). High scores indicate greater anxiety, depression, and general emotional distress. The HADS is a widely used measure and has shown good internal consistency for both subscales (Cronbach's alpha for anxiety = 0.85 and 0.80 for depression) and for the total scale (Cronbach's alpha = 0.89) (Roberts et al., 2001). The HADS is used in CR services as part of routine assessment in the UK (Stafford et al., 2007; Tesio et al., 2014, 2017; British Heart Foundation, 2018). The Cronbach alpha values for the present sample were as follows: 0.81 for anxiety, 0.76 for depression, and 0.84 for the total score.

#### The Metacognitions Questionnaire 30 (MCQ-30; Wells and Cartwright-Hatton, 2004)

The MCQ-30 is a 30-item self-report scale that measures different dimensions of metacognitive beliefs. The questionnaire assess five domains: (i) Cognitive Confidence (e.g., My memory can mislead me at times), (ii) Positive Beliefs about Worry (e.g., Worrying helps me cope), (iii) Negative Beliefs about Uncontrollability and Danger (e.g., When I start worrying I cannot stop, My worrying is dangerous for me), (iv) Cognitive Self-Consciousness (e.g., I pay close attention to the way my mind works), and (v) Need to Control Thoughts (e.g., Not being able to control my thoughts is a sign of weakness). Each domain is a subscale with six items. Respondents rate how much they “generally agree or disagree” with the statements presented on a four-point scale (from 1 to 4). The MCQ-30 has good internal consistency and good test–retest reliability (Wells and Cartwright-Hatton, 2004; Spada et al., 2008; Fergus and Bardeen, 2017). In addition, a five-factor solution of the MCQ-30 was confirmed in medical samples (i.e., cancer and epilepsy) (Cook et al., 2015; Fisher et al., 2016), non-clinical samples (Wells and Cartwright-Hatton, 2004; Spada et al., 2008; Fergus and Bardeen, 2017) and psychiatric disorder samples (Martin et al., 2014; Grötte et al., 2016). Furthermore, a bi-factor solution of the MCQ-30 (i.e., a general factor named metacognitions and five factors representing each subscale) demonstrated good fit in a non-clinical sample (Fergus and Bardeen, 2017).

The Cronbach alpha values for the present sample were as follows: 0.91 for Cognitive Confidence, 0.88 for Positive Beliefs about Worry, 0.83 for Negative Beliefs about Uncontrollability and Danger, 0.81 for Cognitive Self-Consciousness, 0.73 for Need to Control Thoughts, and 0.91 for the Total Score.

### Statistical Analyses

#### Descriptive Statistics

Descriptive statistics included means, standard deviations, and score distributions for the individual CAS-1R items. In addition, mean and standard deviations are reported for the CAS-1R, MCQ-30, and HADS.

#### Measurement Models

The factor structure of the CAS-1R was investigated using confirmatory factor analysis (CFA). If none of the hypothesized factor structures demonstrated an adequate fit to the data, exploratory factor analysis was planned to determine whether a different, non-hypothesized model could be identified.

Four different models for the factor structure of the CAS-1R were hypothesized based on the S-REF model and were compared. The unidimensional model (Figure 1A) was fitted first, principally to provide a baseline for comparison of the more complex models as the expectation was that this model would not fit the data well. In sequence we then fitted the two-factor model, discriminating between coping strategies (6 items) and metacognitions (4 items) (Figure 1B); the three-factor model with a further differentiation between positive (2 items) and negative metacognitive beliefs (2 items) (Figure 1C); and finally the bi-factor model including a general factor on which all items loaded independently of the different specific domains. Factors consisting of only two items are generally not recommended as this can cause problems of model identification and the items may not adequately tap the latent construct (Hair et al., 2010). However, the distinction between positive and negative metacognitive beliefs is theoretically and clinically relevant (Wells and Matthews, 1994, 1996; Wells, 2009).

The hypothesized models were each specified with no correlated errors between the observed variables (Byrne, 2001), with the intention that if no model demonstrated an adequate fit, correlated error terms between observed variables within the same factor would be added based on modification indices (Aish and Joreskog, 1990). The analysis sample of 440 individuals was well in excess of a generally accepted rule of thumb of a minimum sample of 200 for CFA (Kline, 2011; Koran, 2016).

### Model Estimation and Evaluation

AMOS Version 22 (Arbuckle, 2014) was used to conduct CFA within a structural equation modeling framework using maximum likelihood (ML) estimation. Although the CAS-1R items demonstrated non-normal distributions (see below) for which weighted least squares (WLS) is often advocated, simulation studies have demonstrated that ML in fact strongly outperforms WLS (and Generalized Least Squares) under such conditions, including when data is ordinal, and that WLS tends to over-estimate goodness-of-fit (Olsson et al., 2000). The adequacy and parsimony of the models was assessed using a set of commonly-recommended fit statistic indices (Hu and Bentler, 1999; Kline, 2011; Brown, 2015): the Comparative Fit Index (CFI), the Root Mean Square Error of Approximation (RMSEA), the Goodness of Fit Index (GFI), and the Parsimony Goodness of Fit Index (PGFI). We assessed goodness of fit principally on the basis of the CFI and RMSEA, as these indices are least sensitive to sample size and parameter estimates (Hu and Bentler, 1998), using the modern criteria of CFI greater or equal to 0.95 (Hu and Bentler, 1999) and RMSEA <0.08 indicate an acceptable fit and 0.05 a good fit, with an upper 90% confidence limit of 0.1 or less (Browne and Cudeck, 1993). The additional indices were computed to provide a broader picture of model performance and were a GFI value close to 1 and a PGFI above 0.5 which indicate good fit (Mulaik et al., 1989; Hu and Bentler, 1999). We also report the Chi-square statistic, but goodness-of-fit decisions were not based on this as it is known to be sensitive to sample size and to large correlations between factors within the model, making it an unreliable criterion for detecting well-fitting models (Tanaka, 1987). However, the Chi-square difference test was used to statistically compare models according to overall fit, for which it appropriately preserves the alpha-level regardless of sample size (Marsh et al., 2004).

### Assessing Reliability and Validity

The internal consistency of each factor in the resulting model for the CAS-1R was assessed using Cronbach's Alpha and McDonald's Omega coefficient. Alpha is reported, being the commonly accepted standard measure of scale reliability. However, when factor loadings are not equal, alpha underestimates true reliability (Trizano-Hermosilla and Alvarado, 2016) and we therefore also report omega—which is computed directly from the factor loadings—as a generally less biased measure (Trizano-Hermosilla and Alvarado, 2016). Factor uniqueness was assessed using inter-correlations. Convergent and discriminant validity were assessed on the basis of directions and strengths of correlations between the CAS-1R and the MCQ-30, and the HADS. Specifically, a number of relationships were evaluated in line with theoretical expectations: (i) subscales assessing negative metacognitions in the CAS-1R and in the MCQ-30 would correlate positively, and at a higher level than negative CAS-1R metacognitions with MCQ positive beliefs; (ii) subscales assessing positive metacognitions in the CAS-1R and in the MCQ-30 would correlate positively, and at a higher level than positive CAS-1R metacognitions with MCQ negative metacognitions; and (iii) all the CAS-1R subscales would show a positive correlation with the HADS subscales.

### *T*-Tests and Regression Analysis

Independent sample *t*-tests were conducted to explore gender differences in the CAS-1R and the HADS. Although there are no published studies exploring the role of the CAS in CR patients, we hypothesized on theoretical grounds that the CAS-1R would explain anxiety and/or depression above and beyond the variation accounted for by age and gender. To this end, hierarchical regression analysis was conducted. At Step 1, age and gender were entered and at Step 2 all the CAS-1R subscales were included using forced entry.

Assumptions of linearity, homoscedasticity, independence of residuals and the normality of distributed errors were examined to determine whether regression analyses were appropriate (Field, 2013). Regression plots were reviewed to confirm linearity, correlation coefficients between variables were reviewed for multicollinearity, and values of the tolerance and variance inflation factors (VIF) were examined; tolerance values lower than 0.10 or 0.25 are considered a cause of concern (Tabachnick and Fidell, 2001); and VIF values should not exceed 10 (Field, 2013).

## Results

### Descriptive Statistics

There were no missing values for the CAS-1R. The response distributions on each CAS-1R item are given in Table 2. Mean values for items ranged from 23.73 (item 9) to 53.43 (item 1), except for item 6 (*M* = 3.60). Item 6 assesses the use of alcohol to cope with thoughts and feelings, and was the only item substantially skewed, with 88.2% of participants reporting a score of 0 on this item. The lack of score variation on item 6 negatively impacts on estimates of correlation with other items and may be specific to this sub-population. We therefore decided to exclude item 6 from the subsequent structural modeling.

**Table 2:** Descriptive statistics for the CAS-1R Items: Mean, Standard Deviation, frequency and percentage per scale category (*N* = 440).

	**CAS Items**											
		**Item 1**	**Item 2**	**Item 3**	**Item 4**	**Item 5**	**Item 6**	**Item 7**	**Item 8**	**Item 9**	**Item 10**
	M (SD)	53.43 (27.70)	47.30 (30.56)	45.59 (30.08)	44.13 (31.35)	41.02 (29.01)	3.60 (12.77)	40.45 (31.58)	49.66 (34.48)	23.76 (26.56)	42.64 (32.55)
Scale Category Responses: Frequency and Percentage	0	16 (3.6%)	40 (9.1%)	48 (10.9%)	78 (17.7%)	71 (16.1%)	388 (88.2%)	81 (18.4%)	80 (18.2%)	164 (37.3%)	93 (21.1%)
	5	0	1 (0.2%)	0	2 (0.5%)	0	0	0	0	0	1 (0.2%)
	10	33 (7.5%)	41 (9.3%)	35 (8.0%)	31 (7.0%)	38 (8.6%)	17 (3.9%)	40 (9.1%)	24 (5.5%)	58 (13.2%)	27 (6.1%)
	15	1 (0.2%)	2 (0.5%)	4 (0.9%)	1 (0.2%)	1 (0.2%)	1 (0.2%)	1 (0.2%)	0	2 (0.5%)	0
	20	26 (5.9%)	51 (11.6%)	46 (10.5%)	35 (8.0%)	37 (8.4%)	8 (1.8%)	48 (10.9%)	29 (6.6%)	43 (9.8%)	36 (8.2%)
	25	4 (0.9%)	1 (0.2%)	1 (0.2%)	0	0	0	0	0	0	0
	30	45 (10.2%)	37 (8.4%)	41 (9.3%)	25 (5.7%)	36 (8.2%)	13 (3.0%)	42 (9.5%)	34 (7.7%)	43 (9.8%)	34 (7.7%)
	35	1 (0.2%)	0	0	0	1 (0.2%)	0	1 (0.2%)	0	1 (0.2%)	1 (0.2%)
	40	38 (8.6%)	27 (6.1%)	41 (9.3%)	39 (8.9%)	43 (9.8%)	2 (0.5%)	26 (5.9%)	15 (3.4%)	20 (4.5%)	30 (6.8%)
	45	0	1 (0.3%)	1 (0.2%)	0	0	0	0	0	0	1 (0.2%)
	50	68 (15.5%)	64 (14.5%)	62 (14.1%)	62 (14.1%)	87 (19.8%)	3 (0.7%)	67 (15.2%)	52 (11.8%)	57 (13.0%)	62 (14.1%)
	55	0	0	0	0	0	0	0	0	0	0
	60	36 (8.2%)	25 (5.7%)	29 (6.6%)	27 (6.1%)	27 (6.1%)	2 (0.5%)	23 (5.2%)	26 (5.9%)	13 (3.0%)	21 (4.8%)
	65	2 (0.5%)	0	1 (0.2%)	0	0	0	0	2 (0.5%)	0	0
	70	58 (13.2%)	44 (10.0%)	38 (8.6%)	42 (9.5%)	25 (5.7%)	2 (0.5%)	27 (6.1%)	39 (8.9%)	15 (3.4%)	36 (8.2%)
	75	2 (0.5%)	2 (0.5%)	0	2 (0.5%)	2 (0.5%)	0	4 (0.9%)	5 (1.1%)	0	1 (0.2%)
	80	50 (11.4%)	49 (11.1%)	41 (9.3%)	56 (12.7%)	41 (9.3%)	2 (0.3%)	32 (7.3%)	51 (11.6%)	9 (2.0%)	48 (10.9%)
	85	0	0	1 (0.2%)	1 (0.2%)	0	0	2 (0.5%)	1 (0.2%)	0	0
	90	26 (5.9%)	26 (5.9%)	24 (5.5%)	20 (4.5%)	17 (3.9%)	0	16 (3.6%)	35 (8.0%)	4 (0.9%)	23 (5.2%)
	95	0	1 (0.2%)	0	1 (0.2%)	0	0	0	0	0	0
	100	34 (7.7%)	28 (6.4%)	27 (6.1%)	18 (4.1%)	14 (3.2%)	2 (0.5%)	30 (6.8%)	47 (10.7%)	11 (2.5%)	26 (5.9%)

Responses were missing for 20 items on the MCQ-30, and two on the HADS, with no more than two missing responses for any single participant. As the amount of missing data were very small (<0.1% in total) missing values on each scale were replaced with participant means across the completed items.

### CAS-1R Measurement Models

**Figure 2:**
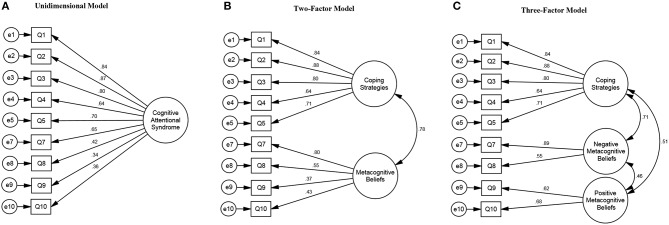
Cognitive Attentional Syndrome Scale-1 Revised (CAS-1R) models and standardized factor loadings (regression weights).

Standardized factor loadings (regression weights) for each of the hypothesized models are presented in Figure 2. Standardized factor loadings on the different models ranged from 0.34 to 0.89.

Goodness-of-fit statistics for each of the measurement models are presented in Table 3. As anticipated, the unidimensional model did not reach our primary criteria (CFI and RMSEA) for adequate fit. The two-factor model showed a significant improvement over the unidimensional solution according to the Chi-squared difference test, and was borderline with regard to our primary fit indices [CFI = 0.953; RMSEA = 0.082 (95% CI 0.066–0.099)]. The three-factor model represented a further significant improvement, with substantially improved fit in terms of CFI and RMSEA and its confidence interval [CFI = 0.988; RMSEA = 0.043 (95%CI 0.022–0.064)]. When attempting to fit a bi-factor model, we experienced problems of identification and negative variance estimates, only solvable by adding additional parameter constraints into the model. Even then, parameter estimates were unstable under different constraint assumptions. We took this as evidence that a bi-factor solution did not fit the data and do not report any further on this model.

**Table 3:** Goodness-of-fit statistics for tested models.

**Model**	**χ^2^**	***df***	***p***	**χ^2^diff^a^, df, *p***	**CFI**	**RMSEA 90% CI [LL-UL]**	**GFI**	**PGFI**
Unidimensional model	151.341	27	<0.001		0.924	0.102 [0.087–0.119]	0.931	0.559
Two-factor model	103.603	26	<0.001	47.74, 1, <0.001	0.953	0.082 [0.066–0.099]	0.951	0.549
Three-factor model	43.920	24	0.008	59.68, 2, <0.001	0.988	0.043 [0.022–0.064]	0.978	0.522

a Reduction in χ

2 *from previous model*.

On the basis of these results we selected the three-factor model as the optimal solution, displaying as it did a good fit on all the criteria and a statistically significant improvement over the two-factor model. The three-factor model discriminates between coping strategies (5 items), negative (2 items), and positive (2 items) metacognitive beliefs; in addition, the correlation between the negative and positive belief factors was 0.46, suggesting that these are reasonably distinct constructs. Therefore, the three-factor model was used for all subsequent analyses.

Patient scores were computed on each sub-scale (factor) as a total score across the included items (rather than applying item weights from the CFA) to reflect how the instrument is used in practice. These sub-scale scores were then used for assessing validity.

### CAS-1R Reliability, Convergent, and Discriminant Validity

Cronbach alpha values were 0.88 for Coping Strategies, 0.65 for Negative Metacognitive Beliefs, and 0.58 for Positive Metacognitive Beliefs. Corresponding omega values were 0.88 for Coping Strategies, 0.70 for Negative Metacognitive Beliefs, and 0.59 for Positive Metacognitive Beliefs. Correlations between the three CAS-1R subscales were all moderate, with the highest being 0.55 between Coping Strategies and Negative Metacognitive Beliefs (Table 4).

**Table 4:** Descriptive statistics and correlations for psychological measures (i.e., CAS-1R, MCQ-30, and HADS).

	**Mean (SD)**	**2**.	**3**.	**4**.	**5**.	**6**.	**7**.	**8**.	**9**.	**10**.	**11**.	**12**.	**13**.
**CAS-1R**													
1. Coping strategies	46.29 (24.51)	0.55	0.37	0.93	0.29	0.55	0.28	0.41	0.45	0.55	0.63	0.48	0.65
2. Negative metacognitive beliefs	45.06 (28.49)	–	0.30	0.75	0.17	0.62	0.24	0.40	0.34	0.49	0.43	0.24	0.40
3. Positive metacognitive beliefs	33.20 (24.96)		–	0.60	0.53	0.25	0.16	0.27	0.37	0.44	0.31	0.10	0.25
**MCQ-30**													
5. Positive beliefs	10.68 (4.49)				–	0.34	0.20	0.40	0.46	0.67	0.36	0.08	0.26
6. Negative beliefs	13.16 (4.65)					–	0.36	0.56	0.55	0.79	0.63	0.34	0.57
7. Cognitive confidence	11.50 (5.05)						–	0.33	0.19	0.61	0.24	0.34	0.33
8. Need for control	11.86 (3.97)							–	0.50	0.76	0.35	0.21	0.33
9. Cognitive self-consciousness	14.62 (4.36)								–	0.75	0.40	0.06	0.27
10. Total	61.81 (16.02)									–	0.55	0.29	0.50
**HADS**													
11. Anxiety	10.32 (3.85)										–	0.45	0.86
12. Depression	8.20 (3.71)											–	0.84
													–

Results relating to assessment of convergent and discriminant validity are summarized in Table 5. As hypothesized, each CAS-1R subscale was found to correlate more highly with similar constructs than with dissimilar constructs. CAS-1R Negative Metacognitive Beliefs correlated highly with MCQ-30 Negative Beliefs and showed a significantly lower correlation with MCQ-30 Positive Beliefs (*r* = 0.62 vs. *r* = 0.17; *p* < 0.001); similarly, CAS-1R Positive Metacognitive Beliefs correlated moderately with MCQ-30 Positive Beliefs and had a significantly lower correlation with MCQ-30 Negative Beliefs (*r* = 0.53 vs. *r* = 0.25; *p* < 0.001). All the CAS-1R subscales were positively correlated with HADS anxiety and HADS depression, though associations with the latter were all lower.

**Table 5:** Summary of investigations of CAS-1R convergent and discriminant validity.

**Hypothesis**	**Correlations**			***z*-score^$^**	***p*-value**
CAS-NEG^*^ correlates more highly with MCQ NEG than with MCQ POS	CAS NEG–MCQ NEG 0.62(a)	CAS NEG–MCQ POS 0.17(b)	MCQ NEG–MCQ POS 0.34(c)	9.72	<0.001
CAS-POS^*^ correlates more highly with MCQ POS than with MCQ NEG	CAS POS–MCQ POS 0.53(a)	CAS POS–MCQ NEG 0.25(b)	MCQ NEG–MCQ POS 0.34(c)	5.82	<0.001
CAS coping/NEG/POS all correlate positively with HADS anxiety	CAS COPING–HADS ANXIETY 0.63				<0.001
	CAS NEG–HADS ANXIETY 0.43				<0.001
	CAS POS–HADS ANXIETY 0.31				<0.001
CAS coping/NEG/POS all correlate positively with HADS depression	CAS COPING–HADS DEPRESSION 0.48				<0.001
	CAS NEG–HADS DEPRESSION 0.24				<0.01
	CAS POS–HADS DEPRESSION 0.10				<0.05

* *CAS NEG, negative metacognitive beliefs; CAS-POS, positive metacognitive beliefs*.

$ *Z-score relating to comparison of (a) with (b) controlling for (c)*.

### *T*-Tests and Regression Analyses

Independent sample *t*-tests exploring gender differences in the CAS-1R subscale scores did not show significant differences. However, gender differences were found to be significant only for HADS-Anxiety scores: males (*M* = 9.81, *SD* = 3.85) and females (*M* = 11.29, *SD* = 3.67); *t*_(320)_ = −3.95, *p* = < 0.001.

Assumptions of linearity, homoscedasticity, independence of residuals, and normally distributed errors were met for regression analyses. The Durbin-Watson test values for all the regression models were all close to 2, indicating that the assumption of independent errors was met (Field, 2013). Tolerance statistics for all regression models were all above 0.62 and the VIF values were all below 2, suggesting collinearity was not a problem (Tabachnick and Fidell, 2001; Field, 2013).

The regression models examined whether as a block the three subscales of the CAS-1R explained variance in anxiety and depression after controlling for age and gender. As shown in Table 6, when predicting HADS-Anxiety and HADS-Depression, the inclusion of the CAS-1R subscales (step 2) was significant and accounted for additional variance: 37% in anxiety and 21% in depression, respectively. At a subscale level, all the three CAS-1R subscales were unique predictors of anxiety; whilst Coping Strategies alone was a significant individual predictor of depression.

**Table 6:** Cognitive Attentional Syndrome Scale-1 Revised (CAS-1R) subscales predicting anxiety and depression symptoms, after controlling for age and gender.

**(A) CAS-1R SUBSCALES PREDICTING SYMPTOMS OF ANXIETY**						
		**Step 1**			**Step 2**		
	***β***	**95% CI**	***p***	***β***	**95% CI**	***p***
Age	−0.15	[−0.24, −0.59]	0.001	0.005	[−0.07, 0.08]	0.904
Gender (female)	0.41	[0.22, 0.60]	<0.0001	0.25	[0.10, 0.41]	**0.001**
*R*^2^; *F*; *p*-value	0.056; 12.98; <0.0001					
CAS-1R coping strategies				0.52	[0.43, 0.61]	**<0.0001**
CAS-1R negative metacognitive beliefs				0.10	[0.02, 0.19]	**0.018**
CAS-1R positive metacognitive beliefs				0.10	[0.02, 0.17]	**0.015**
*R*^2^; *R*^2^ change; *F* for change in *R*^2^; *p*-value				0.425; 0.369; 92.80; **<0.0001**		
**(B) CAS-1R SUBSCALES PREDICTING SYMPTOMS OF DEPRESSION**						
	**Step 1**			**Step 2**		
	***β***	**95% CI**	***p***	***β***	**95% CI**	***p***
Age	−0.12	[−0.22, −0.03]	0.009	−0.01	[−0.10, 0.08]	0.075
Gender (female)	0.20	[0.00, 0.39]	0.049	0.06	[−0.12, 0.24]	0.512
*R*^2^; *F*; *p*-value	0.022; 5.00; 0.007					
CAS-1R coping strategies				0.06	[−0.12, 0.24]	**<0.0001**
CAS-1R negative metacognitive beliefs				−0.03	[−0.13, 0.07]	0.569
CAS-1R positive metacognitive beliefs				−0.07	[−0.16, 0.02]	0.103
*R*^2^; *R*^2^ change; *F* for change in *R*^2^; *p*-value				0.233; 0.211; 39.75; **<0.0001**		

*Bold values represent a significant p-value*.

## Discussion

The assessment and monitoring of change in purported underlying causal mechanisms of anxiety and depression in patients with medical conditions is a priority for evaluating and interpreting psychological treatment outcomes. This is the first study investigating the factor structure of a measure assessing the CAS in a sample of cardiac patients with mild to severe symptoms of anxiety and/or depression. The measure is grounded in the S-REF model (Wells and Matthews, 1994, 1996) which proposes that the CAS is a key construct in explaining the maintenance of psychological disorders.

Results of the CFA showed that the best fit for the CAS-1R data in cardiac patients experiencing emotional distress corresponded to a three-factor model distinguishing between unhelpful coping strategies (e.g., worry, rumination, avoidance), negative and positive metacognitive beliefs, supporting the value in separating these constructs. This separation of factors maps neatly onto the focus of metacognitive therapy that aims to increase patient awareness of CAS processes, bring them under control and challenge negative and positive metacognitive beliefs (Wells, 2009).

This study found positive associations between the CAS-1R and anxiety and depression symptoms, which is consistent with previous findings using the CAS-1 in clinical (Fergus et al., 2013) and non-clinical samples (Fergus et al., 2012; Fergus and Scullin, 2017). These positive relationships were also found among samples with physical conditions, i.e., patients with cancer (McNicol et al., 2013; Cook et al., 2015; Fisher et al., 2017) and multiple sclerosis (Heffer-Rahn and Fisher, 2015).

The results of the regression analyses provide evidence that the three components of CAS-1R are significant statistical predictors of anxiety among cardiac patients after controlling for age and gender. The CAS-1R was also a predictor of depression symptoms, although the only significant contributing factor was coping strategies. This could be related to the sample being more anxious than depressed.

The alcohol use item of the CAS-1R was very highly skewed and was removed from the analysis. Participants' answers to this item may reflect CR patients being asked to stop unhealthy behaviors, such as smoking and drinking, and some responses may have been aspirational rather than actual. It is anticipated that this item may perform differently in other populations and it may retrieve valuable information in other samples.

The CFA yielded a good fit for one of the CAS-1R hypothesized model, i.e., the three-factor model. Internal consistency was excellent for the coping strategies factor, acceptable for negative metacognitions, but well below the conventional threshold of 0.70 for positive metacognitions. The latter two factors each included just two items, which may be contributing to lower internal consistency. However, derived subscales scores showed good convergent and discriminant validity with the MCQ-30 subscales (i.e., positive beliefs about worry and negative beliefs about uncontrollability and danger), suggesting that these subscales have practical utility in spite of this.

The present study provides support for the use of the multiple dimensions of the CAS-1R in research settings and its continued use in clinical settings. Findings suggest that psychological treatments for anxiety and depression in cardiac patients should target both unhelpful thinking styles and coping strategies and metacognitive beliefs. Generalization of the psychometric properties of the CAS-1R to populations with different mental health diagnoses and other psychical illnesses warrants further research.

### Strengths and Limitations

Strengths of this study include a reasonably large sample of more than 400 participants used to test the theoretical models, no missing data on the CAS-1R, and only a very small amount of missing data for the MCQ-30 and the HADS (<0.01%). However, some limitations warrant discussion. Data were not collected to examine test-retest reliability of the CAS-1R, meaning that this area remains unexplored and should be considered in future research. The measure is intended to be a state measure that is sensitive to variation in the CAS, but a limitation at the present time is a lack of data on responsivity of the measure. It is important to highlight that two of the factors are measured by just two items which may provide limited coverage of these constructs. If more comprehensive assessment of negative and positive metacognitive beliefs is required, the MCQ-30 could be used alongside the CAS-1R.

### Conclusion

This study investigated the factor structure and some of the psychometric properties of a measure of the CAS. Findings provide preliminary evidence supporting a theoretically consistent and well-fitting three-factor solution. Given these findings it is recommended that the measure be used to evaluate change in putative maintenance factors during the course of psychological therapy for anxiety and depression in cardiac samples. The use of the CAS-1R measure in future research could help to enhance understanding of psychological processes involved in treatment response and maintenance of emotional distress in cardiac patients and other populations.

## Data Availability

The datasets generated for this study are available on request to the corresponding author.

## Ethics Statement

Ethical approval for the PATHWAY programme has been granted by the NHS Research Ethics Committee, UK (References: 15/NW/0136, 16/NW/0786). All patients provided written informed consent.

## Author Contributions

AW designed the CAS-1R and CF, DR, and AW designed the study. CF, LC, and RA recruited, consented and administered the baseline measures to participants. CF and CH conducted the statistical analysis supervised by DR and AW. CF drafted the initial manuscript. DR and AW revised the manuscript. All authors contributed and agreed the final draft.

### Conflict of Interest Statement

AW is the developer of metacognitive therapy and a co-director of the Metacognitive Therapy Institute. The remaining authors declare that the research was conducted in the absence of any commercial or financial relationships that could be construed as a potential conflict of interest.
